# Two Benthic Diatoms, *Nanofrustulum shiloi* and *Striatella unipunctata*, Encapsulated in Alginate Beads, Influence the Reproductive Efficiency of *Paracentrotus lividus* by Modulating the Gene Expression

**DOI:** 10.3390/md19040230

**Published:** 2021-04-17

**Authors:** Francesca Glaviano, Nadia Ruocco, Emanuele Somma, Giuseppe De Rosa, Virginia Campani, Pasquale Ametrano, Davide Caramiello, Maria Costantini, Valerio Zupo

**Affiliations:** 1 Department of Marine Biotechnology, Stazione Zoologica Anton Dohrn, Villa Comunale, 80121 Napoli, Italy; francesca.glaviano@szn.it (F.G.); nadia.ruocco@szn.it (N.R.); emanuele.somma@szn.it (E.S.); pas.ametrano@studenti.unina.it (P.A.); 2 Department of Biology, University of Naples Federico II, Complesso Universitario di Monte Sant’Angelo, Via Cinthia 21, 80126 Napoli, Italy; 3 Department of Life Sciences, University of Trieste, 34127 Trieste, Italy; 4 Department of Pharmacy, University of Naples Federico II, 80131 Naples, Italy; giuseppe.derosa2@unina.it (G.D.R.); virginia.campani@unina.it (V.C.); 5 Department of Research Infrastructures for Marine Biological Resources, Marine Organisms Core Facility, Stazione Zoologica Anton Dohrn, Villa Comunale, 80121 Napoli, Italy; davide.caramiello@szn.it

**Keywords:** encapsulation, microalgae, modulated genes, sea urchin development

## Abstract

Physiological effects of algal metabolites is a key step for the isolation of interesting bioactive compounds. Invertebrate grazers may be fed on live diatoms or dried, pelletized, and added to compound feeds. Any method may reveal some shortcomings, due to the leaking of wound-activated compounds in the water prior to ingestion. For this reason, encapsulation may represent an important step of bioassay-guided fractionation, because it may assure timely preservation of the active compounds. Here we test the effects of the inclusion in alginate (biocompatible and non-toxic delivery system) matrices to produce beads containing two benthic diatoms for sea urchin *Paracentrotus lividus* feeding. In particular, we compared the effects of a diatom whose influence on *P. lividus* was known (*Nanofrustulum shiloi*) and those of a diatom suspected to be harmful to marine invertebrates, because it is often present in blooms (*Striatella unipunctata*). Dried *N. shiloi* and *S. unipunctata* were offered for one month after encapsulation in alginate hydrogel beads and the larvae produced by sea urchins were checked for viability and malformations. The results indicated that *N. shiloi*, already known for its toxigenic effects on sea urchin larvae, fully conserved its activity after inclusion in alginate beads. On the whole, benthic diatoms affected the embryogenesis of *P. lividus*, altering the expression of several genes involved in stress response, development, skeletogenesis and detoxification processes. Interactomic analysis suggested that both diatoms activated a similar stress response pathway, through the up-regulation of *hsp60*, *hsp70*, *NF-κB*, *14-3-3 ε* and *MDR1* genes. This research also demonstrates that the inclusion in alginate beads may represent a feasible technique to isolate diatom-derived bioactive compounds.

## 1. Introduction

Dietary uptake of organic and inorganic compounds influence the physiology [1,2], ecology [3] and the population dynamics [4] of several marine grazers. Besides pollution and other anthropogenic influences [5], which trigger specific effects on the physiology of planktonic and benthic consumers, algae are known to produce various defense metabolites, characterized by a range of activities, from simple repulsive effects [6] to highly toxigenic effects against grazers [7] or their progeny [8,9].

The environmental impact of algal metabolites is often evaluated by forcing invertebrates to graze on given plants [10], or exposing small aquatic animals to the volatile organic compounds constitutively produced by algae, to record their behavioral reactions [11,12,13]. However, bioassay-guided fractionation is often required to isolate the active compounds triggering these reactions, and further elucidate their molecular structure, for biotechnological applications [14]. The administration of algal extracts and fractions may reveal some constraints, because it must be gathered through suitable feeds supplemented with polar or non-polar compounds. In seawater, some lipophilic compounds are stably stored in feeds, until their complete consumption by invertebrates [15]. Hydrophilic compounds, in contrast, are easily dispersed in the water after administration and cannot reach their target consumers if not ingested immediately [16].

A variety of encapsulation techniques may be adopted to stably conserve the secondary metabolites previously fractionated or isolated, and guarantee their correct vehiculation to the target organism. In particular, the development of encapsulation improves the efficiency of screening methods and offers a fast, simple and reliable tool (e.g., multiplexed screening methods) to test the effect of algal metabolites on their consumers. To this end, carboxylated microspheres [17] were used to immobilize various toxins on their surface and were easily vehiculated through compound feeds. An alternative approach is the adoption of microencapsulated feeds [18,19] since they may consistently and almost stably preserve various types of compounds avoiding contact with the seawater. Microencapsulated feeds, indeed, are commonly added to dry feeds and they can be stored in refrigerators maintaining their stable shapes. When they are added to humid feeds, or directly dispersed in the water (in the case of filter feeders or very small organisms), the lipidic capsules may slowly degrade and this may represent an issue, if experiments are supposed to last more than a few days.

For this reason, marine polysaccharides such as alginates and carragenates may be adopted to produce particles of variable size, able to preserve and deliver active compounds [20]. Marine polysaccharides are a large and quite complex group of macromolecules sharing some interesting biological properties. The field of marine polysaccharides is constantly evolving over the last decades, thanks to a wide variety of compounds extracted from marine organisms that are tailored for these applications. Red macroalgae are mostly used for the extraction of polysaccharides, but alternative sources may be green and brown seaweeds, as well as marine prokaryotes. The use of seaweed-derived alginates embodies an additional advantage when feeds are prepared for grazers, because their chemical properties are normally suitable for the physiology of various consumers. Moreover, these compounds are widely recognized for their taste and toughness, besides their well-known non-cytotoxic characteristics, biodegradability and biocompatibility. For example, algae-produced hydrogels, based on cross-linked polysaccharides, are often employed for drug delivery systems and tissue engineering. Polysaccharides derived from marine organisms may also be employed to produce superabsorbent/superporous hydrogels [20].

Marine polysaccharides are increasingly used for nutraceutical and cosmaceutical applications, particularly as tools for the incorporation of bioactive agents [21]. It is worth noting that sodium alginate, in combination with polyacrylics such as either poly(acrylic acid), poly(acrylamide) or both, forms interpenetrating networks that give rise to superabsorbent and superporous hydrogels [22], occasionally adopted to direct active compounds towards specific animal targets [23]. Alginate dressings maintain a physiologically moist microenvironment that is feasible for containing a variety of active compounds. Alginate is a naturally occurring polysaccharide of organic acids (guluronic and mannuronic), abundant in nature as a structural component of brown algae, but it may also be obtained from soil bacteria. Although the microencapsulation technique was initially developed for oral delivery of proteins and other compounds, which are quickly degraded in the acid gastric environment, this technique was demonstrated to produce interesting results for bioassay-guided isolation of natural compounds [24]. In addition, sodium alginates may be modified with amine or acid moieties to optimize their efficiency for drug delivery applications. These modifications permit modulation of the erosion time, the release rates, and even their adhesion to specific substrates [21]. For example, using super-hydrophobic surfaces, polymer particles may be produced [25] allowing the loading of compounds into spherical structures (microspheres) with huge encapsulation efficiency [26]. Several benthic diatoms are commonly ingested by sea urchins, because they produce dense epiphytic layers on seagrasses and seaweeds, commonly grazed by these echinoderms. However, artificial feeds (e.g., agar blocks including specific diatoms) are needed to test the effect of individual microalgae on the reproductive physiology of sea urchins. Algae must be included when still fresh, to avoid rapid deterioration in the aqueous environment, prior to be ingested. Conserved algae (e.g., frozen diatoms) cannot be used for this purpose, because the loss of structural properties of their siliceous shells would immediately produce leaking of active compounds. This limitation imposes complex research procedures and various experimental limits.

Various diatoms contribute to toxic plankton blooms, including *Thalassiosira* spp., *Nitzschia* spp. and *Striatella* spp. [27] and their effects on planktonic consumers were extensively investigated [1]. Several harmful microalgae, known to produce biotoxins and cause fish and invertebrate deaths, were identified and documented. However, similar diatom genera are present in the periphytic benthos too [28], although their effects on benthic consumers are scarcely investigated. In particular, *S. unipunctata* is a benthic diatom often found in the gut content of various grazers and filter feeders [29] and its toxigenic properties are still to be investigated. This species appears in the list of most abundant taxa during harmful micro-phytoplankton blooms [30] but its direct effects on filter-feeders and grazers were never documented.

Previous investigations indicated that live marine benthic diatoms, *Nanofrustulum shiloi*, included in agar blocks produce clear physiological effects in the sea urchin, *Paracentrotus lividus* [8]. However, due to the several issues mentioned above, investigations may exclusively progress by using diets based on either frozen diatoms, diatom fractions, or both. Accordingly, this investigation attempted the administration of frozen benthic diatoms, *N. shiloi*, whose physiological effects were already demonstrated by simple inclusion of live diatoms, as a control for the inclusion in alginate hydrogel beads, never attempted before. In addition, *Striatella unipunctata* was tested in alginate beads because its physiological effects on sea urchins were never investigated. Consequently, we compared the known activity of *N. shiloi,* here considered as a positive control, to the effects of *S. unipunctata* and the seaweed *Ulva rigida*, adopted as control diet. Both diatoms may be seasonally abundant on the leaf surface of the seagrass, *Posidonia oceanica* [31,32,33,34], which represents a preferred food item for *P. lividus*. The toxigenic effects of encapsulated diatoms on sea urchin progenies were evaluated by morphological observations of plutei and gene expression analyses.

## 2. Results

### 2.1. Species Identification by Morphological and Molecular Analyses

The observation under SEM and optical microscopy revealed that the benthic diatom tested in the present study belonged to the araphid pennate species, *S. unipunctata* Agardh 1832 (Figure S1), whose first description was reported as *Fragilaria unipunctata* Lyngbye 1819. The diatom is about 70 μm in length, flat and rectangular in shape and with truncated corners. *S. unipunctata* was easily recognizable through its valve ornamentation that consists of septate girdle bands covering all the surfaces of frustules. Under light microscopy, it was possible to observe the typical reticulate chloroplasts and the mucilaginous stalk, which is secreted through a corner of the frustule allowing for attachment to the bottom (Figure S1).

Molecular data totally agreed with the morphological characterization obtained by spicule observations. BLASTn alignments revealed about 99% pairwise-sequence similarity to four strains of *S. unipunctata* ribosomal 18S RNA gene (accession numbers: JX419383.1, AB430609.1, AF525666.1 and HQ912643.1) with 100% query cover (Figure S2).

### 2.2. Diatom’s Encapsulation

As reported in Table S1, the alginate beads without diatoms showed a mean diameter of about 3.8 mm, after preparation. The encapsulation of diatoms led to a very slight reduction of the mean diameter of ~3.7 mm. As expected, dehydration strongly reduced the size of the beads resulting in 1.3 mm and 1.2 mm for alginate beads and diatoms/alginate beads, respectively.

### 2.3. Effects of Feeding Tests on Sea Urchin Progeny

#### 2.3.1. Fertilization/Cleavage Rates and Embryo Development

**Figure 1:**
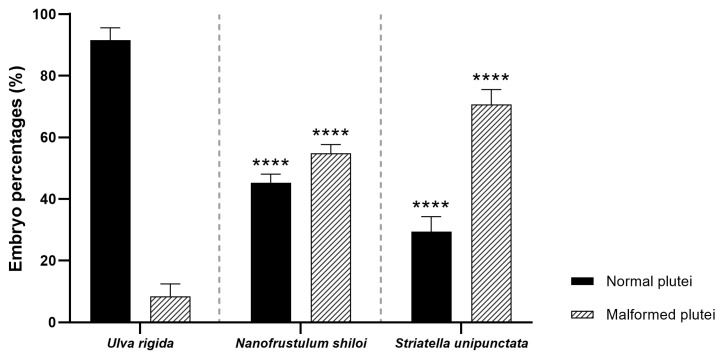
Percentages of normal and malformed plutei derived from sea urchins fed with the control diet (*U. rigida*), *N. shiloi* and *S. unipunctata* encapsulated in alginate beads. One-way ANOVA followed by Tukey post hoc for multiple comparisons: **** *p* < 0.0001. Pairwise comparison was reported between control (*U. rigida* diets) vs. samples treated with *N. shiloi* and *S. unipunctata*.

After a one month of feeding with *U. rigida*, *N. shiloi* and *S. unipunctata*, eggs and sperms were collected prior to fertilization. The percentage of fertilized eggs and embryos at the two-cell stage resulted in 100% for all samples under analysis. These data were similar to those obtained for sea urchins collected from the wild and spooned at the beginning of the experiment (T_0_). The morphological observations performed on sea urchin embryos 48 h post-fertilization (hpf) revealed that both diatoms induced several malformations affecting the apex and the arms [35] (Figure 1 and Figure S3). Firstly, *N. shiloi* exerted similar effects to those previously reported in Ruocco et al. [8], with a percentage of abnormal plutei of about 55% (*p* > 0.05) (Figure 1).

This result confirmed that diatom encapsulation, within the alginate beads, did not cause additional effects on embryonic development. Furthermore, *S. unipunctata* induced a significant increase in malformed plutei (70%), being statistically different from the percentages obtained in sea urchins fed on *U. rigida* (about 10%, *p* < 0.0001) (Figure 1). Interestingly, the toxicity of *S. unipunctata* diets on sea urchin progenies was found significantly higher than *N. shiloi* treated individuals (*p* < 0.0001) (Figure 1).

#### 2.3.2. Gene Expression by Real Time (RT)-qPCR

**Figure 2:**
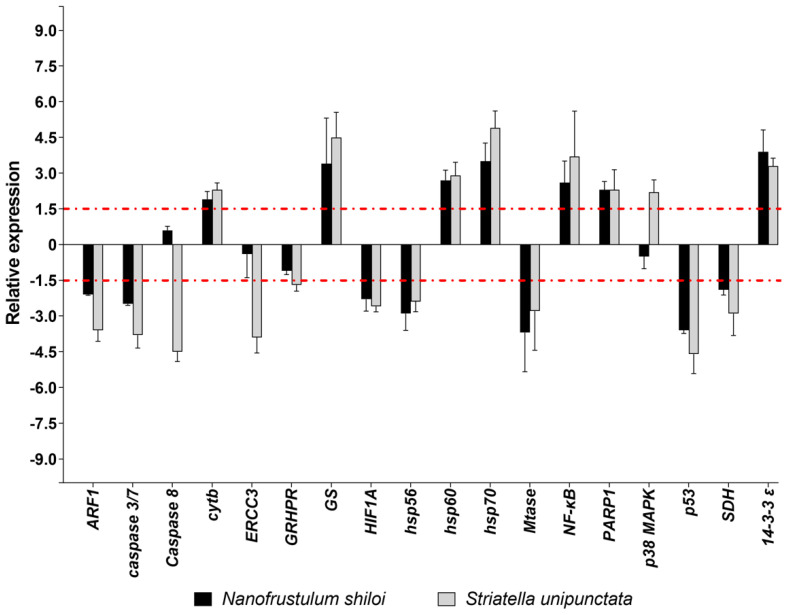
Fold changes of stress response genes in plutei from sea urchin adults fed with *N. shiloi* (black bar) and *S. unipunctata* (gray bar) encapsulated in alginate beads. The dotted red line represents the cut-off (1.5). Values are reported as the average fold changes ± SD (*n* = 3). Statistical differences were evaluated by nonparametric Mann–Whitney test. *p*-values < 0.05 were considered significant.

Among the eighteen genes belonging to stress response, *N. shiloi* altered the expression of several genes, except for *Caspase 8*, *ERCC3*, *GRHPR* e *p38 MAPK* (Figure 2). On the contrary, *S. unipunctata* confirmed to induce the strongest effects by changing the expression levels of all stress genes under analysis (Figure 2). On the whole, both diatoms had a lot of common targets, down-regulating *ARF1*, *caspase 3/7*, *HIF1A*, *hsp56*, *Mtase*, *p53* and *SDH*, and up-regulating *cytb*, *GS*, *hsp60*, *hsp70*, *NF-κB*, *PARP1* and *14-3-3 ε*. Moreover, *S. unipunctata* solely induced a variation of four stress genes with a decrease of *Caspase 8*, *ERCC3*, *GRHPR* and an increase of *p38 MAPK* (Figure 2).

**Figure 3:**
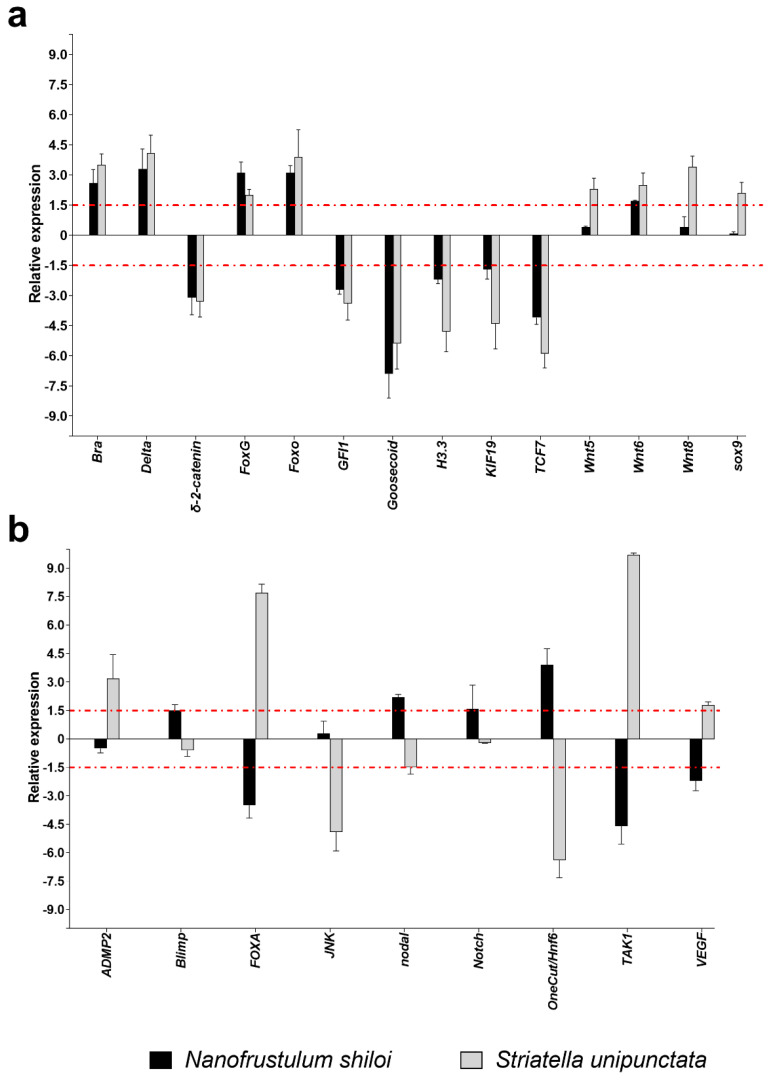
Fold changes of development and differentiation genes in plutei from sea urchin adults fed with *N. shiloi* (black bar) and *S. unipunctata* (gray bar) encapsulated in alginate beads where (**a**) the two diatoms induced the same gene expression (up- or down-regulation for both diatoms) or (**b**) different gene expression (up- in one diatom and down-regulation in the other diatom). The dotted red line represents the cut-off (1.5). Values are reported as the average fold changes ± SD (*n* = 3). Statistical differences were evaluated by nonparametric Mann–Whitney test. *p*-values < 0.05 were considered significant.

The functional class grouping development and differentiation processes was the most represented, with 28 genes analyzed. In particular, *N. shiloi* and *S. unipunctata* induced the up-regulation of *Bra*, *Delta*, *FoxG*, *Foxo* and *Wnt6*, and the down-regulation of *δ-2-catenin*, *GFI1*, *Goosecoid*, *H3.3*, *KIF19* and *TCF7* (Figure 3a).

In contrast, different results were detected in the cases of *FOXA*, *TAK1* and *VEGF* genes, whose expression increased with *S. unipunctata* and decreased with *N. shiloi*; and *nodal* and *OneCut/Hnf6*, revealing the opposite effect (Figure 3b). Moreover, benthic diatoms individually switched on the expression of *Blimp* and *Notch* (*N. shiloi*), and *ADMP2* (*S. unipunctata*) (Figure 3b).

**Figure 4:**
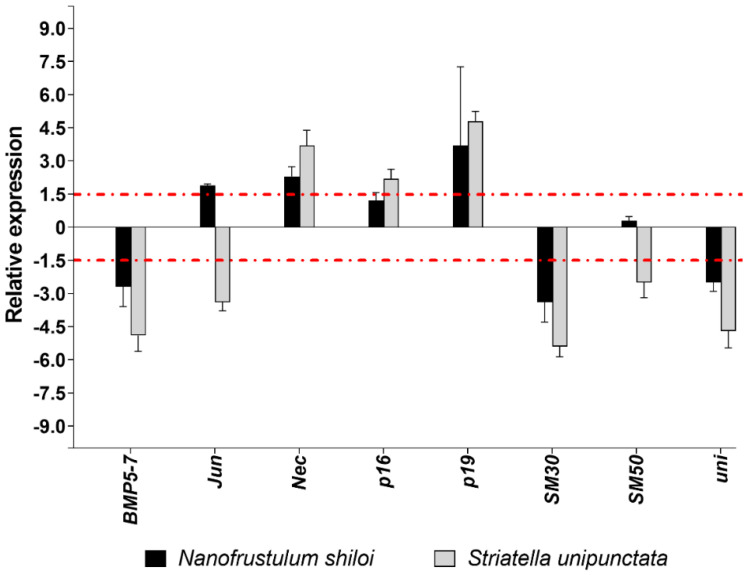
Fold changes of skeletogenesis genes in plutei from sea urchin adults fed with *N. shiloi* (black bar) and *S. unipunctata* (gray bar) encapsulated in alginate beads. The dotted red line represents the cut-off (1.5). Values are reported as the average fold changes ± SD (*n* = 3). Statistical differences were evaluated by nonparametric Mann–Whitney test. *p*-values < 0.05 were considered significant.

Concerning the eight genes involved in the skeletogenesis of sea urchin plutei, *N. shiloi* and *S. unipunctata* had five common targets. Specifically, both diatoms up-regulated Nec and p19 and down-regulated *BMP5-7*, *SM30* and *uni* (Figure 4).

Different molecular effects were detected for *Jun*, since both the up- (*N. shiloi*) and down-regulation (*S. unipunctata*) was found (Figure 4). *S. unipunctata* also altered the expression of *p16* (up-regulation) and *SM50* (down-regulation) genes (Figure 4).

**Figure 5:**
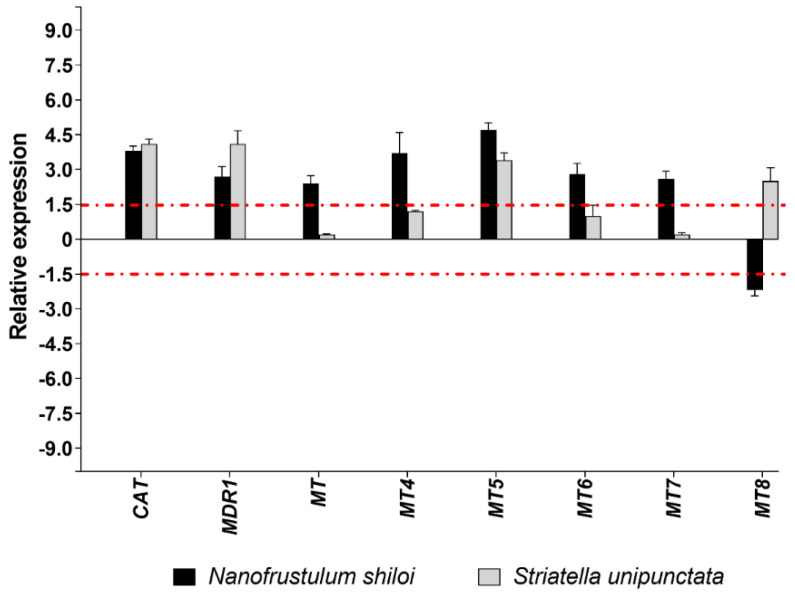
Fold changes of detoxification genes in plutei from sea urchin adults fed with *N. shiloi* (black bar) and *S. unipunctata* (gray bar) encapsulated in alginate beads. The dotted red line represents the cut-off (1.5). Values are reported as the average fold changes ± SD (*n* = 3). Statistical differences were evaluated by nonparametric Mann–Whitney test. *p*-values < 0.05 were considered significant.

*N. shiloi* was able to change the expression of all genes belonging to detoxification processes, while half of them were significantly altered by *S. unipunctata*. Benthic diatoms shared the up-regulation of three genes, namely, *CAT*, *MDR1* and *MT5* (Figure 5).

Furthermore, the *MT8* gene was up-regulated by *S. unipunctata* and down-regulated by *N. shiloi* (Figure 5). Interestingly, *N. shiloi* was able to up-regulate all methallothioneins under study (*MT*, *MT4*, *MT5*, *MT6* and *MT7*), except for the *MT8* gene (Figure 5).

## 3. Discussion

In the present work we evaluated the harmful effects of diatom diets on the sea urchin P. lividus by encapsulating two diatom species, *N. shiloi* and *S. unipunctata*, in alginate hydrogel beads, combining morphological and molecular approaches.

### 3.1. Effects of Feeding Tests by Morphological Observations

Our results showed that *N. shiloi* induced several malformations in sea urchin progenies with a percentage of about 60% of aberrant plutei (*p* < 0.0001), confirming our previous investigations [8] (Figure 1; see also Figure S3). Moreover, *S. unipunctata* was also toxic to sea urchins, inducing stronger effects (75% malformed plutei) than *N. shiloi*, with a high statistical significance (*p* < 0.0001; Figure 1). Interestingly, *N. shiloi* was previously found to produce several toxic compounds, named oxylipins, that together with other unknown compounds, could induce such negative effects [36]. The accumulation of these bioactive secondary metabolites could be responsible for reducing gamete quality, as well as interfering with the fertilization and embryonic development processes, as demonstrated by Ruocco et al. [36] through Pearson correlation analysis. For this reason, we encapsulated diatoms in alginate beads to preserve the active molecules and avoid any dispersion into the surrounding water. In fact, the encapsulation of extremely sensitive compounds, such as proteins, vitamins and dehydrated extracts, within alginate hydrogel beads protect them from the external environment [37,38]. Furthermore, calcium-alginate beads were used in this work since they represent a biocompatible and non-toxic delivery system, which is very easy to prepare through cost-effective procedures without applying high temperatures [39,40].

### 3.2. Effects of Feeding Tests on Gene Pathways

Concerning the molecular investigation of 62 genes belonging to stress response, development and differentiation, skeletogenesis and detoxification processes, benthic diatoms were able to switch on almost all genes under analysis (Figure 2, Figure 3, Figure 4 and Figure 5; Table S2). These molecular results corroborated our observation under light microscopy showing that feeding on benthic diatoms induced several malformations in sea urchin plutei (Figure 1).

Among the 62 genes analyzed, more than half were positively or negatively altered by both diatoms, revealing a similar variation in gene expression and a huge number of shared targets (Figure 2, Figure 3, Figure 4 and Figure 5). On the other hand, a few genes were impaired differently, particularly those involved in development and differentiation events, such as *FOXA*, *nodal*, *OneCut/Hnf6*, *TAK1* and *VEGF* (Figure 3b). These results could indicate that benthic diatoms affect some common molecular pathways by changing the normal biological mechanisms, which, in turn, generate aberrant progenies in sea urchins.

**Figure 6:**
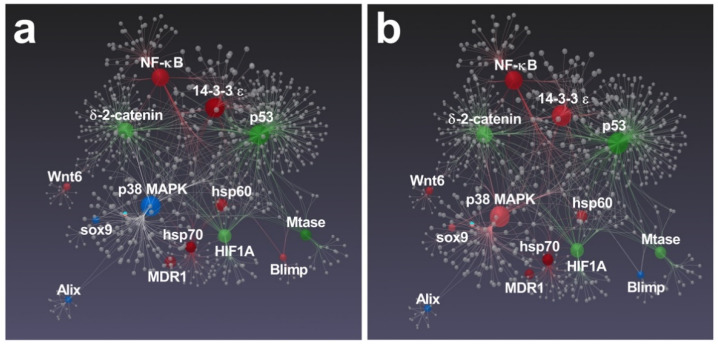
Gene network performed by STRING interactome of protein–protein interactions. Correlations with high confidence score cut-off (900) were reported. Among functionally correlated genes, those with up (red), down (green) and unchanged (blue) expression affected by *N. shiloi* (**a**) and *S. unipunctata* (**b**) were reported. Color shading depends on fold-change values. Gray spheres represent additional connections.

Interestingly, several genes followed by RT-qPCR in the present study were previously found to be functionally interconnected [41,42,43]. In particular, Varrella et al. [41] showed that both diatoms altered the expression of all genes belonging to the network, except for *Alix*, whose relative expression was not significant (Figure 6).

The majority of these correlated genes were similarly affected by both diatoms with the only exception of Blimp, which was up-regulated in *N. shiloi* (Figure 6a) and *sox9/p38 MAPK*, whose expression was up-regulated in *S. unipunctata* (Figure 6b).

On the whole, a similar stress response pathway, mediated by *hsp60*, *hsp70*, *NF-κB*, *14-3-3 ε* and *MDR1*, was activated since this group of interconnected genes was significantly up-regulated after the feeding with both benthic diatoms. Some of these genes were previously proposed to be involved in the same molecular pathway in response to UV-B radiation and, recently, after the exposure of polystyrene nanoparticles [44,45,46]. Moreover, after analyzing gene expression data, we found that both diatoms induced the up-regulation of *NF-κB* signaling, which represent a fundamental cascade for the activation of the innate immune system following several stress events that causes DNA damage [47].

**Figure 7:**
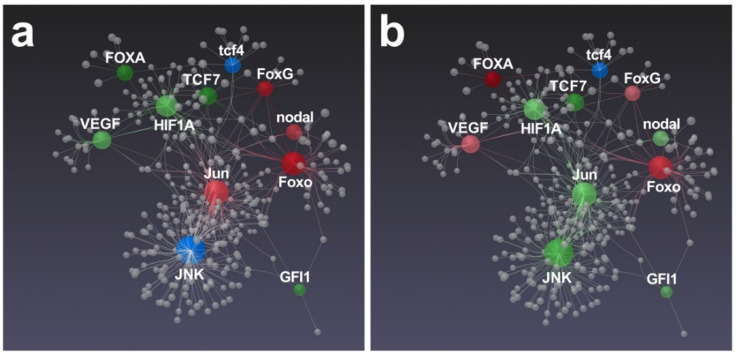
Gene network performed by STRING interactome of protein–protein interactions. Correlations with high confidence score cut-off (900) were reported. Among functionally correlated genes, those with up (red), down (green) and unchanged (blue) expression affected by *N. shiloi* (**a**) and *S. unipunctata* (**b**) were reported. Color shading depends on fold change values. Gray spheres represent additional connections.

Concerning the gene network proposed by Ruocco et al. [42], in which mostly development and skeletogenesis genes were involved, interesting differences were brought to light (Figure 7).

In fact, a key transcription factor that controls skeletogenesis in sea urchins (*Jun*) [48] was significantly up-regulated by *N. shiloi* (Figure 7a). The connected *Foxo*, *FoxG* and *nodal* genes were also up-regulated, suggesting that a molecular pathway probably mediated through these genes could be activated by this diatom (Figure 7a). On the contrary, *S. unipunctata* induced the down-expression of *Jun* and *JNK* (Figure 7b) that, in turn, could trigger the aberrations in the sea urchin embryos, since both genes were reported to exert key roles in skeletogenesis and development [48,49]. In addition, *S. unipunctata* was able to increase the relative expression of the *VEGF* gene (Figure 7b), which is demonstrated to be involved in sea urchin spiculogenesis [50]. However, recent data on sea urchin gene regulatory networks (GRN) reported that hypoxia-induced stress is able to perturb the expression of *HIF1A*, *nodal* and *VEGF* pathways, inducing severe effects on the structure of larval skeletons [51]. This is also the case with our results, reporting a significant variation of these latter genes following diatom feeding, that might explain the anomalies in sea urchin plutei observed under light microscopy (Figure 1; see also Figure S3). In fact, we found a significant down-regulation of *HIF1A*, which is known to limit the expression of *nodal* to the ventral side of sea urchin embryos for the activation of down-stream transcription factors [52]. The dysregulation of *HIF1A* and *nodal* genes could be responsible for such malformations (Figure S3).

**Figure 8:**
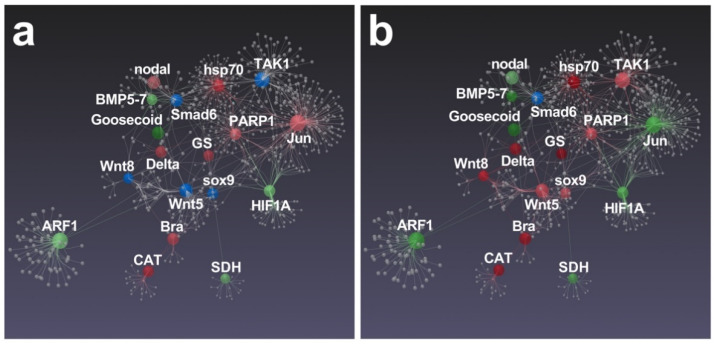
Gene network performed by STRING interactome of protein–protein interactions. Correlations with high confidence score cut-off (900) were reported. Among functionally correlated genes, those with up (red), down (green) and unchanged (blue) expression affected by *N. shiloi* (**a**) and *S. unipunctata* (**b**) were reported. Full color shows greater fold change values. Gray spheres represent additional connections.

Significant differences between *N. shiloi* and *S. unipunctata* were also found in the gene correlation analysis performed by Esposito et al. [43], since only *S. unipunctata* was able to target all connected genes, with the exception of only *Smad6* (Figure 8b).

Again, we found that *Jun*, strongly connected with several genes [48], was specifically up-regulated by *N. shiloi*. This gene was probably at the basis of huge molecular cascade activated by this diatom (Figure 8a). Within this pathway, we also noticed the involvement of *hsp70*, *PARP1*, *GS*, *Delta*, *nodal*, *Bra* and *CAT* genes, since mRNA levels significantly increased in sea urchin plutei deriving from adults fed with *N. shiloi* (Figure 8a). Then, *S. unipunctata* was able to up-regulate a great number of genes that displayed a connection with several genes in the same biological pathway, such as *TAK1*, *hsp70*, *PARP1*, *GS*, *sox9*, *Wnt5*, *Wnt8*, *Delta*, *Bra* and *CAT* (Figure 8b).

More specifically, PARP1 level increased in sea urchin plutei, deriving from adults fed on both benthic diatoms (Figure 8).

The up-regulation of genes involved in DNA repair may be due to a potential genotoxic effect triggered by unknown or known [36] (e.g., oxylipins) compounds that were supplied through diatom diets [53]. Moreover, a significant activation of the *CAT* gene was observed (Figure 8), probably induced by the accumulation of reactive oxygen species (ROS) in sea urchin eggs. The expression alteration of this gene might be connected to the clear aberrations of sea urchin embryos (Figure 1; see also Figure S3), since the mitochondrial redox signaling via H_2_O_2_ is well known to perturb *nodal* expression and the following oral-aboral axis specification [54].

Analyzing RT-qPCR data, we detected the alteration of *nodal*, *Delta*, *Bra* and *Goosecoid*, which are normally required for the early specification of ectodermal tissues [55]. Interestingly, *nodal* was found to influence the expression of BMP signaling and some dorsal marker genes, such as *Wnt5*, *Wnt8*, *smad6*, together with *sox9*, which is a transcription factor involved in left-right asymmetry specification [55,56,57]. These experimental evidences were also corroborated by our results, since benthic diatoms induced the opposite regulation of *nodal* gene (up-regulated in *N. shiloi* and down-regulated in *S. unipunctata*) and, in turn, a different expression pattern in down-stream effectors (Figure 8).

Overall, the majority of genes, whose expression was modified by diatom feeding, are involved in the specification of ectodermal, endodermal and mesodermal cell fates during sea urchin embryo development. Despite several connections still being largely unknown due to the high complexity of molecular responses, some of them were experimentally demonstrated to join the same GRN [55,56,57,58,59,60,61,62,63].

## 4. Materials and Methods

### 4.1. Ethics Statement

*Paracentrotus lividus* (Lamarck) adults were collected from a site in the Bay of Naples that is not privately owned or protected in any way, according to Italian legislation (DPR 1639/68, 19 September 1980, confirmed on 10 January 2000). Field studies did not include endangered or protected species. All experimental procedures on animals followed the guidelines of the European Union (directive 2010/63/EU).

### 4.2. Isolation of S. unipunctata and Culturing

*S. unipunctata* was isolated from samples of *P. oceanica* leaves collected in the field from the meadows located in Lacco Ameno (Island of Ischia, Gulf of Naples, Italy). Once in the laboratory, the lamina of each leaf was rinsed with filtered seawater (FSW) and then gently scraped with a glass slide in order to collect the epiphytic communities. The diatoms of interest were isolated under inverted microscope (Leica Microsystems), through sequential transfer of single cells, by means of a micromanipulator (Leica Microsystems). The isolated diatom was deployed in multi-well plates filled with sterile seawater in order to obtain axenic cultures. The monoclonal strains thus obtained were gently renovated under a laminar flow hood and cultured in 12-multiwell plates with Guillard’s f*/2* medium (Sigma-Aldrich, Milan, Italy) and kept in a thermostatic chamber at 18 °C, with a 12 h:12 h light:dark photoperiod. Light was provided by Silvania GroLux (Osram Sylvania Inc., Wilmington, Massachusetts, USA) at 140 μE∙m^−2^∙s^−1^ irradiance.

### 4.3. Species Characterization

#### 4.3.1. Morphological Analysis of Frustules

Diatom identification was firstly performed using a morphological approach, through the analysis of the frustule ultrastructure on images captured through a scanning electron microscopy (SEM, JEOL 6700F, JEOL Ltd., Akishima, Tokyo, Japan). Diluted monoclonal cultures were collected in graduated Pyrex glass tubes and cleaned through acid treatment by adding H_2_SO_4_ (96%) and HNO_3_ (65%). After six washing cycles with distilled water, the pH was evaluated using litmus paper. Once neutral pH was reached, the cleaned samples were mounted on stubs and sputter-coated with platinum for SEM observations.

#### 4.3.2. Molecular Identification of Diatom Species

Cell cultures were collected from the multi-well plates and concentrated by centrifugation for 20 min at 1800 relative centrifugal force (rcf) at 4 °C for 15 min, then frozen in liquid nitrogen until use. About 20 mg of pellet was treated with lysis buffer containing 2% cetyltrimethylammonium bromide (CTAB) and 2-mercaptoethanol (2-ME, Sigma-Aldrich). Then, DNA extraction was performed according the protocol reported in Ruocco et al. [8].

The total amount of DNA extracted was estimated by measuring the absorbance at 260 nm; purity was calculated using 260/280 and 260/230 nm ratios, using a NanoDrop spectrophotometer (ND-1000 UV-vis Spectrophotometer; NanoDrop Technologies, Wilmington, DE, USA). The integrity of DNA was evaluated by agarose gel electrophoresis.

Polymerase chain reactions (PCRs) were performed using specific primers targeting the 18S rRNA region (528F/1055R [8,64]). Sequences were submitted to the NCBI (National Center for Biotechnology Information) database through Basic Local Alignment Search Tool (BLASTn available at https://blast.ncbi.nlm.nih.gov/Blast.cgi, accessed on 1 February 2021 [65]) in order to identify the best hits with higher percentage of identity. In addition, PCR fragments were aligned to all 18S sequences found using the software MultiAlin (available at http://multalin.toulouse.inra.fr/multalin/, accessed on 1 February 2021 [66]).

### 4.4. Diatom Encapsulation in Alginate Beads

Monoclonal cultures of *N. shiloi* and *S. unipunctata* were grown as reported above. For experimental purposes, massive cultures were inoculated in 14 cm Petri dishes containing 100 mL of f/2 medium. At the end of the exponential phase, cells were counted under a Neubauer chamber and biomass was evaluated as logC (quantity of intracellular carbon in picograms) = −0.541 + 0.811 × logV (cell volume in μm^3^) [8,67]. The same biomass previously used for the diatom *N. shiloi* in Ruocco et al. [8] was then collected and lyophilized.

Dried diatoms were then encapsulated in alginate hydrogel beads [38,39,40]. Alginate beads were prepared as described by Zhang et al. [37], with some modifications. Briefly, a 2% alginate solution (*w*/*v*) was prepared by dissolving sodium alginate powder in deionized water under magnetic stirring for about 3 h. Then, freeze-dried diatoms were dispersed in the sodium alginate solution at the predetermined concentration. Contemporarily, a calcium chloride solution (5% *w*/*v*) was prepared by melting calcium chloride powder in double distilled water under magnetic stirring at room temperature. Diatoms/alginate solution was then poured, drop by drop, into the corresponding 5% calcium chloride solution under continuous and gently stirring. The resulting alginate beads were then collected, washed with deionized water and dried for 48 h at room temperature. Dry beads were stored at 4 °C before use.

The diameter of alginate beads was measured after preparation, in the hydrated form, and immediately after dehydration, to evaluate whether diatoms were perfectly encapsulated. Measurements were carried out by analysis of digital images of alginate beads, using open-source software, Image J (Java 1.8.0_112). The mean diameters were calculated as the average values over three different measurements (*n* = 3).

### 4.5. Feeding, Gametes Collection, Evaluation of Fertilization/Cleavage Success and Detection of Abnormal Plutei

Alginate beads were included into a 2% agar substrate and used as food for sea urchins with a daily rate of 1 g of agar per sea urchin [8,68]. Twenty adult (12 females and 8 males) *P. lividus* were reared in each experimental tank with a continuous flow-through system [8] and fed with *U. rigida* (3 control replicates) and the 2 benthic diatoms tested (3 replicates for each species). After 1 month of feeding, eggs and sperm were collected. Eggs were washed three times with filtered seawater (FSW) and kept in FSW until use. Concentrated “dry” sperm was collected and kept undiluted at +4 °C until use. Eggs were fertilized utilizing sperm-to-egg ratios of 100:1. Fertilized eggs were kept at 20 °C in a thermostatic chamber on a 12 h:12 h light:dark cycle. After 48 hpf, morphological malformations were determined for at least 100 sea urchin plutei from each female (fixed in 0.5% glutaraldehyde) using a light microscope (Zeiss Axiovert 135TV, Carl Zeiss, Jena, Germany).

### 4.6. Molecular Analysis on Sea Urchin Plutei

About 5000 eggs (in 50 mL of FSW) from each female fed on *U. rigida* and the two benthic diatoms were collected and fertilized. Embryos were then collected at 48 hpf by centrifugation at 1800 rcf for 10 min in a swing out rotor at 4 °C. Embryos were placed in at least 10 volumes of the RNAlater (Qiagen, Hilden, Germany), and then frozen in liquid nitrogen. Samples were kept at −80 °C until use.

Total RNA was extracted using Aurum Total RNA Mini Kit (Bio-Rad, Hercules, CA, USA), according to the manufacturer’s instructions for RNA-seq experiments. For each sample, 600 ng of total RNA was retrotranscribed with an iScript cDNA synthesis kit (Bio-Rad, Milan, Italy), following the manufacturer’s instructions.

Gene expression analysis was performed on three biological replicates. The levels of each gene were followed by real time-qPCR. The relative expression ratios were calculated from quantification cycles (Cq) through an efficiency (E) corrected calculation method (E_target_^∆Cq target (Mean Control–Mean Sample)^/E_reference_^∆Cq reference (Mean Control–Mean Sample^) [69,70], using REST software (Version No., Relative Expression Software Tool, Weihenstephan, Germany). *Ubiquitin* [71] and *18S rRNA* [46,72] were selected as reference genes, since no variation was assessed during the embryonic development of the sea urchin and between control and treated samples. Values larger than 1.5-fold were considered significant.

To elaborate the 3 gene networks previously published in literature [41,42,43], an interactomic analysis was performed by NetworkAnalyst 3.0 software [73] available at https://www.networkanalyst.ca/ (accessed on 1 February 2021), using STRING interactome of protein–protein interactions [74]. Human orthologs of selected genes were used to compute the network analysis. The most significant relations among genes (confidence score cut-off = 900) displaying experimental evidence were highlighted.

### 4.7. Statistical Analysis

Data-sets were analyzed by D’agostino and Pearson normality test to ensure that values were normally distributed. Statistical differences of normal and malformed embryos among testing groups were evaluated by one-way analysis of variance (ANOVA), followed by Tukey’s post hoc test for multiple comparisons (*n* = 15). Regarding RT-qPCR data, a nonparametric Mann–Whitney test was applied to ∆Cq (Cq gene of interest—Cq reference) values between treated and control samples (*n* = 3).

*p*-Values larger than 0.05 were considered significant. Statistical analyses were performed using GraphPad Prism Software (version 9.00 for Windows, GraphPad Software, La Jolla, CA, USA, www.graphpad.com, accessed on 1 February 2021).

## 5. Conclusions

In the present work, we demonstrated that microencapsulation of diatoms for feeding purposes could be an efficient experimental system to vehicle toxic sensitive compounds, since we observed a clear negative effect on sea urchin progenies. The two benthic diatoms tested in the present work (*N. shiloi* and *S. unipunctata*), induced similar malformations, which were confirmed by the alteration of several genes involved in stress response, development, skeletogenesis and detoxification processes. The two diatoms shared several molecular targets thus supporting the hypothesis that they could probably activate the same molecular pathways. However, among those genes, previously found to be functionally interconnected [41,42,43], slight expression differences were observed. This result suggests that benthic diatoms under analysis did not necessarily trigger the same biological cascade, particularly in skeletogenesis and development processes.

## Data Availability

Not applicable.

## Supplementary Materials

The following are available online at https://www.mdpi.com/article/10.3390/md19040230/s1, Figure S1: Images of *S. unipunctata* under the optical microscope (a) and SEM (b,c). The diatom shows a big chloroplast in the middle of the cell (a), a frustule with rectangular shape and truncated corners (b), a mucilaginous stalk (b) and characteristic ornamental striae on the surface of each valve (c). Scale bars = 15 µm (a), 10 µm (b) and 1 µm (c). Figure S2: Alignment of *S. unipunctata* to the four annotated 18S rRNA sequences found in the BLASTn search with accession numbers JX419383.1 (a), AB430609.1 (b), AF525666.1 (c) and HQ912643.1 (d). Forward and reverse primers were highlighted by a green and sky-blue rectangle, respectively; Figure S3: Images of sea urchin embryos under optical microscope. Normal (a) and malformed (b,c) plutei were reported (Zeiss Axiovert135TV microscope, 10×/0.30 magnification/numerical aperture). Scale bar: 50 µm; Table S1: Diameter in millimeters (mm) of alginate beads before and after dehydration; Table S2: Fold change values of 62 genes belonging to stress response, development and differentiation, skeletogenesis and detoxification processes analyzed by RT-qPCR. Up-regulated genes and down-regulated genes were highlighted in red and blue, respectively.
