# Supplementary material for: Two Benthic Diatoms, Nanofrustulum shiloi and Striatella unipunctata, Encapsulated in Alginate Beads, Influence the Reproductive Efficiency of Paracentrotus lividus by Modulating the Gene Expression
Source: Mar Drugs. 2021 Apr 17;19(4):230. doi: 10.3390/md19040230 (PMC8074093; doi:10.3390/md19040230)
Supplement: Supplementary file 1 [file marinedrugs-19-00230-s001.zip › marinedrugs-1167344-supplementary.pdf]

Two benthic diatoms, *Nanofrustulum shiloi* and *Striatella unipunctata*, encapsulated in alginate beads, influence the reproductive efficiency of *Paracentrotus lividus*

Francesca Glaviano[1,#], Nadia Ruocco[1,#], Emanuele Somma[1,#], Giuseppe De Rosa[2], Virginia Campani[2], Pasquale Ametrano[1], Maria Costantini[1,*], Valerio Zupo[1,*]

[1]*Department of Marine Biotechnology, Stazione Zoologica Anton Dohrn, Villa Comunale, 80121 Napoli, Italy*

[3]*Department of Pharmacy, University of Naples Federico II, Naples, Italy*

[#]These authors contributed equally to this work

*Authors to whom correspondence should be addressed: maria.costantini@szn.it, vzupo@szn.it, Tel.: +39-081-583-3315; Fax: +39-081-764-1355

Figure S1. a. Images of *S. unipunctata* under the optical microscope (a) and SEM (b-c). The diatom shows a big chloroplast in the middle of the cell (a), a frustule with rectangular shape and truncated corners (b), a mucilaginous stalk (b), and characteristic ornamental striae on the surface of each valve (c). Scale bars= 15 µm (a), 10 µm (b), 1 µm (c).

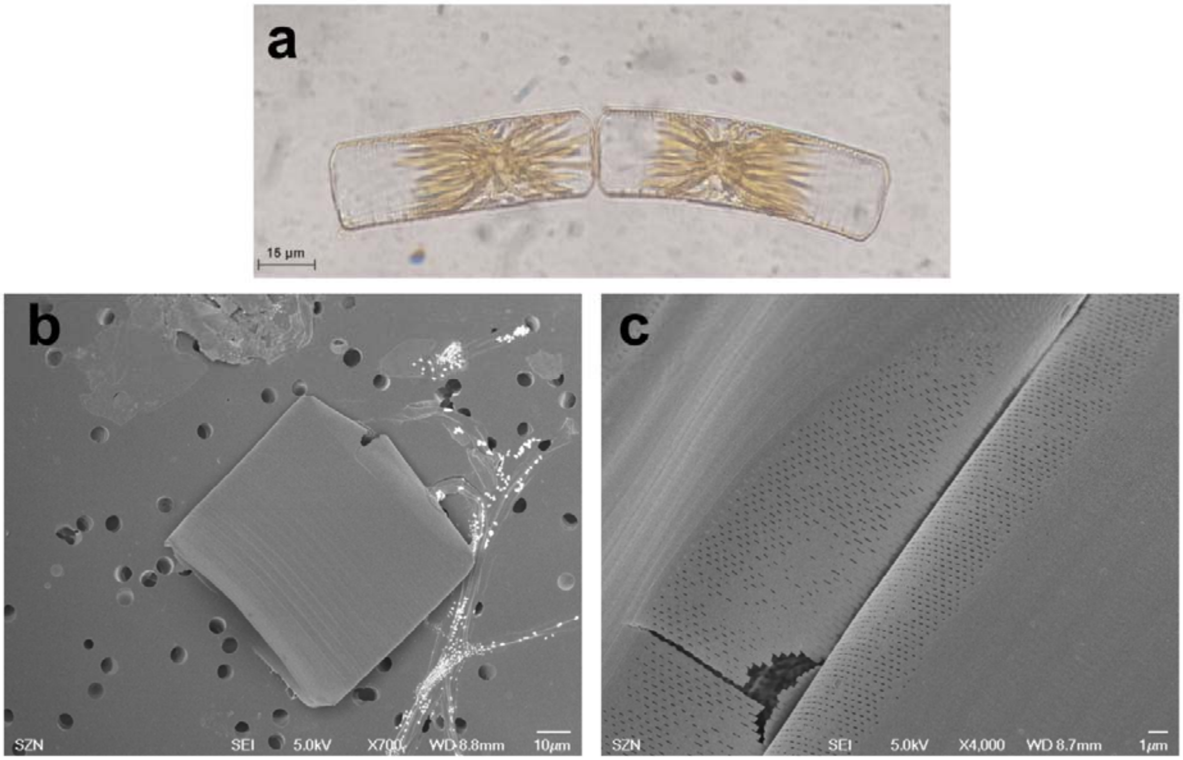

Figure S2. Alignments of *S. unipunctata* to the four annotated 18S rRNA sequences found in BLASTn search with accession numbers JX419383.1 (a), AB430609.1 (b), AF525666.1 (c) and HQ912643.1 (d). Froward and reverse primers were highlighted by a green and sky blue rectangle, respectively.

a

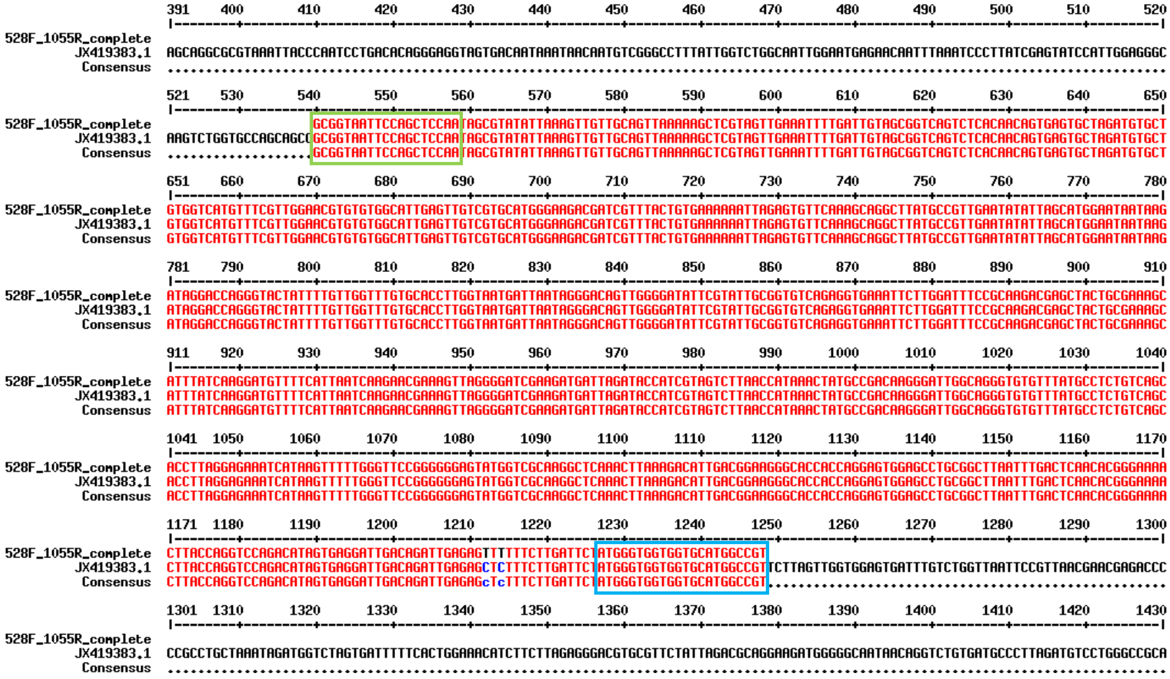

b

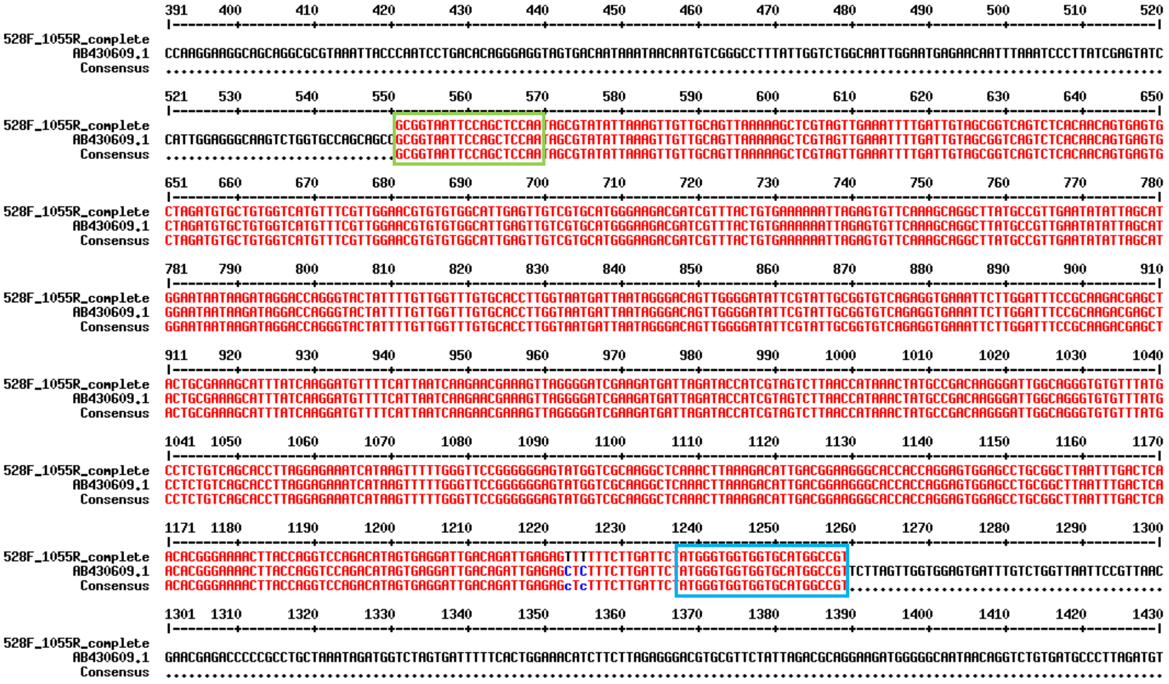

c

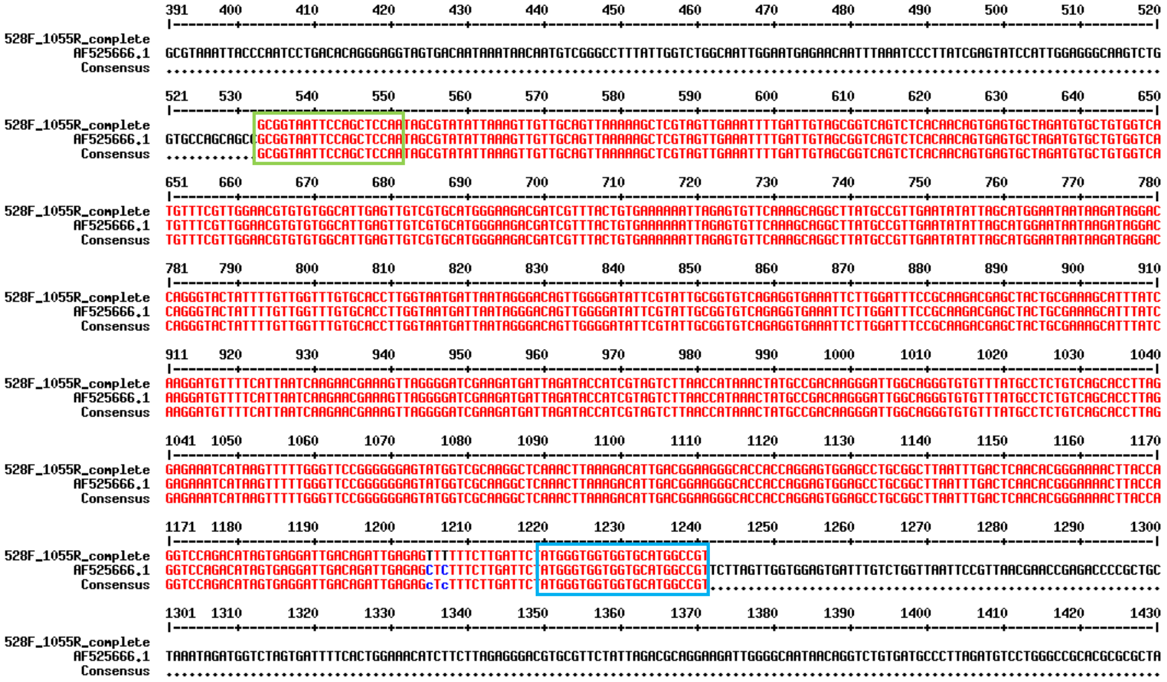

d

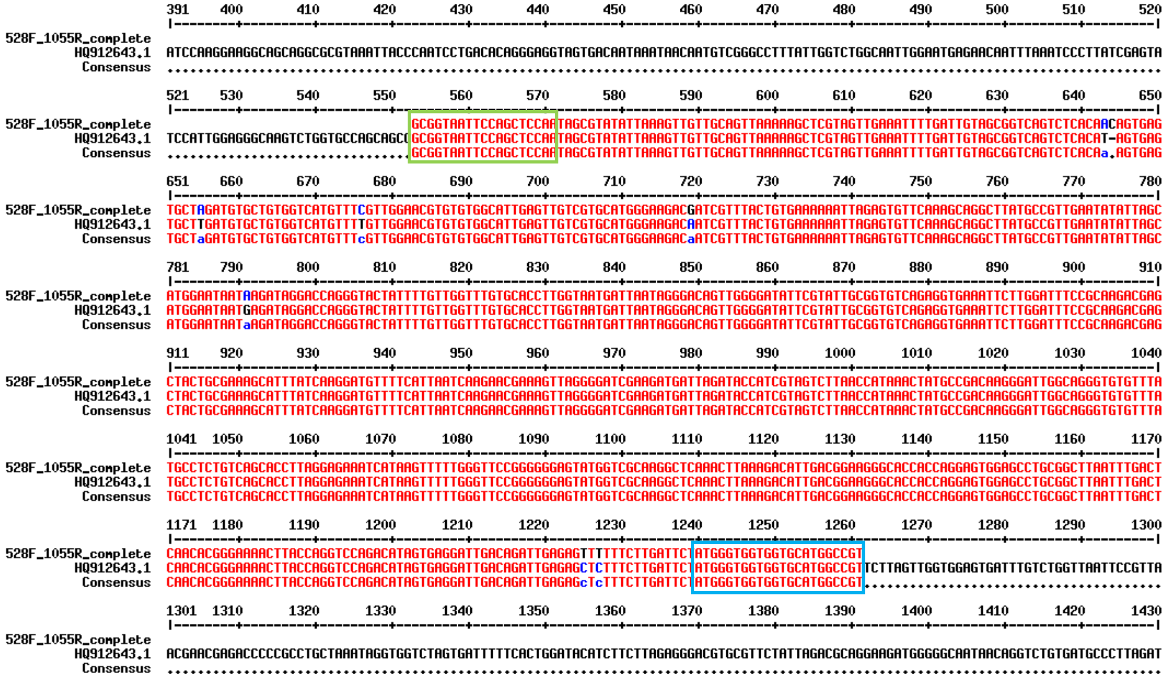

Figure S3. Images of sea urchin embryos under optical microscope. Normal (a) and malformed (b-c) plutei were reported (Zeiss Axiovert135TV microscope, 10×/0.30 magnification/numerical aperture). Scale bar: 50 μm.

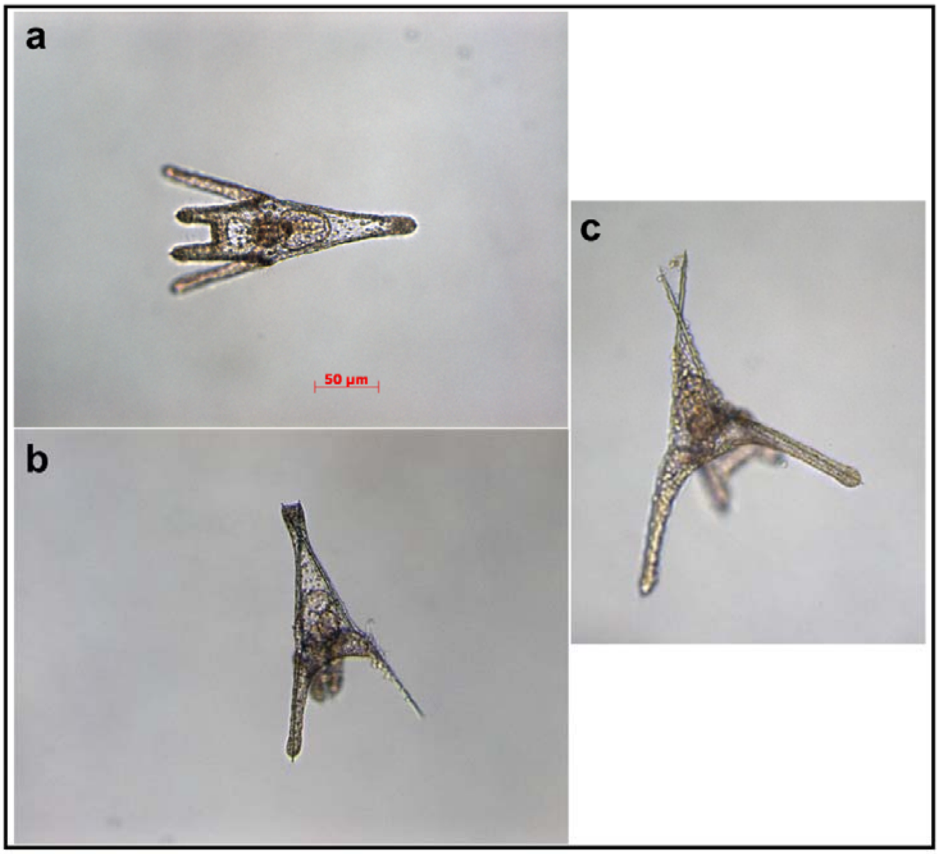

Table S1. Diameter in millimeters (mm) of alginate beads before and after dehydration.

	**Diameter (mm)**	
	Hydrated form	Dehydrated form
Alginate beads	3.8 ± 0.2	1.3 ± 0.1
Diatoms/alginate beads	3.7 ± 0.2	1.2 ± 0.1

Table S2. Fold change values of sixty-two genes belonging to stress response, development and differentiation, skeletogenesis and detoxification processes analyzed by RT-qPCR. Up-regulated genes and down-regulated genes were highlighted in red and blue, respectively.

		N. shiloi	***S. unipunctata***
Stress	*ARF1*	-2.1	-3.6
	caspase 3/7	-2.5	-3.8
	CASP8	0.6	-4.5
	cytb	1.9	2.3
	ERCC3	-0.4	-3.9
	GRHPR	-1.1	-1.7
	GS	3.4	4.5
	HIF1A	-2.3	-2.6
	hsp56	-2.9	-2.4
	hsp60	2.7	2.9
	hsp70	3.5	4.9
	MTase	-3.7	-2.8
	NF-kB	2.6	3.7
	PARP	2.3	2.3
	p38 MAPK	-0.5	2.2
	p53	-3.6	-4.6
	SDH	-1.9	-2.9
	14-3-3 ε	3.9	3.3
Development/ Differentiation	*ADMP2*	-0.5	3.2
	Alix	0.6	0.5
	Blimp	1.5	-0.6
	BP10	0.3	0.5
	BRA	2.6	3.5
	DELTA	3.3	4.1
	δ-2-catenin	-3.1	-3.3
	FOXA	-3.5	7.7
	FoxG	3.1	2.0
	Foxo	3.1	3.9
	GFI1	-2.7	-3.4
	GOOS	-6.9	-5.4
	hat	-0.4	-0.7

	H3.3	-2.2	-4.8
	JNK	0.3	-4.9
	KIF19	-1.7	-4.4
	nodal	2.2	-1.5
	NOTCH	1.6	-0.2
	OneCut	3.9	-6.4
	SMAD6	0.9	0.8
	sox9	0.1	2.1
	TAK1	-4.6	9.7
	tcf4	0.7	0.1
	TCF7	-4.1	-5.9
	VEGF	-2.2	1.8
	Wnt5	0.4	2.3
	Wnt6	1.7	2.5
	Wnt8	0.4	3.4
Skeletogenesis	*BMP5-7*	-2.7	-4.9
	C-jun	1.9	-3.4
	Nec	2.3	3.7
	p16	1.2	2.2
	p19	3.7	4.8
	SM30	-3.4	-5.4
	SM50	0.3	-2.5
	uni	-2.5	-4.7
Detoxification	*CAT*	3.8	4.1
	MDR1	2.7	4.1
	MT	2.4	0.2
	MT4	3.7	1.2
	MT5	4.7	3.4
	MT6	2.8	1.0
	MT7	2.6	0.2
	MT8	-2.2	2.5
